# Clinical Profile and Predictors of Mortality in Neonates Born With Non-Immune Hydrops Fetalis: Experience From a Lower-Middle-Income Country

**DOI:** 10.7759/cureus.17830

**Published:** 2021-09-08

**Authors:** Vinod K Hasija, Adnan Mirza, Waqar H Khowaja, Sidra Asif, Muhammad Sohail Salat, Shabina Ariff, Khalil Ahmad

**Affiliations:** 1 Pediatrics-Neonatal Medicine, Aga Khan University Hospital, Karachi, PAK; 2 Pediatrics-Neonatal Medicine, Limerick University Maternity Hospital, Limerick, IRL; 3 Neonatal Fellowship Program/Neonatal Intensive Care Unit, Aga Khan University Hospital, Karachi, PAK; 4 Pediatrics and Child Health, Aga Khan University Hospital, Karachi, PAK; 5 Pediatrics, Aga Khan University Hospital, Karachi, PAK

**Keywords:** hydrops fetalis, pleural effusion, neonate, ascites, generalized anasarca

## Abstract

Introduction

Hydrops fetalis (HF) is a life-threatening condition in which a fetus has an abnormal collection of fluid in the tissue around the lungs, heart, abdomen, or under the skin. Based on its pathophysiology, it is classified into immune and non-immune types. With the widespread use of anti-D immunoglobulin, non-immune HF has become more common, with an incidence of one in 1,700-3,000 live births. A multitude of fetal diseases with various causes can lead to non-immune HF. Due to the recent advances in prenatal diagnostic and therapeutic interventions together with improved neonatal intensive care, the diagnosis and subsequent management of HF have been refined. However, HF is still associated with a high mortality rate. A recent assessment of the literature found that there is a lack of data on prognostic variables in neonates with HF from low- and middle-income countries. In light of this, we sought to establish the etiologic causes, predictors of mortality, and eventual fate of newborns born non-immune HF at the Aga Khan University Hospital, Karachi during the 10-year period spanning January 2009-December 2019 in this retrospective analysis.

Methodology

For this study, we collected data from the computerized database and patient record files at the hospital on all infants with non-immune HF. Demographic data, postnatal interventions, clinical and laboratory findings, outcomes, and the results of comparison between HF patients who died and those who survived were analyzed.

Results

The incidence of non-immune HF at our hospital was 0.62/1,000 live births during the period under study, with 33 newborn babies diagnosed with non-immune HF from a total of 53,033 live-born deliveries. An etiologic factor was discovered in 17 (51.5%) neonates with non-immune HF while 16 (48.4%) were classified as those with unidentified etiology. The most common causes were cardiovascular and genetic syndromes, which resulted in 100% mortality. The overall mortality rate was 67%. The need for mechanical ventilation, surfactant therapy, and prolonged hospitalization were identified as independent risk factors of mortality.

Conclusion

Our study proves that the need for mechanical ventilation [moderate to severe hypoxic respiratory failure (HRF)] and prolonged hospitalization are strong predictors of poor outcomes in neonates with non-immune HF. Therefore, severe hydrops causing significant mortality can be anticipated based on the patients' respiratory status and the need for escalated oxygen support.

## Introduction

Hydrops fetalis (HF) is a well-recognized fetal condition, and it is defined as the presence of extracellular fluid either in at least two fetal body compartments or one body compartment in the presence of skin edema (>5-mm thickness). Ascites, pleural and pericardial effusions, and anasarca are all symptoms of pathologic buildup of extracellular fluid in the skin or bodily compartments. Most instances of HF have a non-immune etiology due to the decline in rhesus isoimmunization. According to the literature, non-immune HF accounts for roughly 76-87% of all cases of HF, while its incidence varies from one in 1,700-3,000 live births [1-3]. A significant number of fatal diseases with various causes can lead to non-immune HF. The principal pathophysiology of non-immune HF is consistent with abnormal fluid transportation between plasma and tissues, which are either geared by an increase in hydrostatic pressure and capillary permeability or the reduction of the plasma osmotic pressure or lymphatic flow [4]. Recent breakthroughs in prenatal diagnostic and therapeutic measures, as well as enhanced postnatal intensive care, have led to significant advances in the diagnosis and subsequent treatment of non-immune HF. On the other hand, the mortality rate is still very high [5-6]. Recent research demonstrates that there is a lack of evidence on prognostic variables in newborn babies with non-immune HF, which is mostly dependent on the underlying etiology [7-10].

In this study, we aimed to determine the etiologic factors, the predictors of mortality, and the eventual outcomes in newborns born with non-immune HF at a tertiary care referral center in Pakistan over a period of 10 years.

## Materials and methods

This was a retrospective hospital study carried out at the neonatal intensive care unit (NICU) of the Agha Khan University Hospital in Karachi, Pakistan on newborns born with non-immune HF at the facility during the 10-year period spanning from January 2009 to December 2019. Institutional Ethics Review Committee (IERC) permission was obtained, with an issued certificate number (2020-5270-13945). The study only included live-born infants who survived in the delivery room with or without the requirement for neonatal resuscitation. Our non-immune HF cohort was defined as those having generalized skin edema with pathologic collection of serous fluid in one or more fetal body compartments or the presence of extracellular fluid in at least two fetal body compartments. Immune HF was classified based on clinical and laboratory results (maternal and neonatal blood group, evidence of hemolysis, and Coombs test). Postnatal diagnostic and therapeutic interventions, demographic data, APGAR score at the fifth minute, postnatal therapies such as mechanical ventilation and surfactant therapy, causative factors of non-immune HF, and mortality were all evaluated for each neonate.

All categorical variables such as gender, consanguinity, diagnosis, and mode of delivery were presented as numbers and percentages. Continuous variables such as gestational age, weight, and height were expressed as mean ± standard deviation (SD), while the duration of mechanical ventilation and hospitalization were presented as median. The parametric test was applied when data were normally distributed whereas non-parametric tests were applied when data were skewed. The Kolmogorov-Smirnov test was used to check the assumption of normal distribution. The chi-square/Fisher's exact test was applied according to whether the cell expected frequency was smaller than 5, and it was also applied to determine the significant association between categorical variables. The unadjusted odds ratio was calculated to find out the association and potential risk factors related to mortality. Multivariate logistic regression analysis by backward elimination method was applied to determine the most significant risk factors/predictors of the risk of mortality. Due to the small sample size, we could not attain variables in the final model at a 5% level of significance. Therefore, we attained variables at a 10% level of significance. All data were analyzed using Stata version 16 (StataCorp, College Station, TX).

As per our NICU policy, a baseline workup, including complete blood count with peripheral smear, Coombs test and TORCH [toxoplasmosis, rubella cytomegalovirus (CMV), herpes simplex, and HIV] screening, echocardiography, ultrasound, and X-ray chest and abdomen, was performed for all neonates with HF.

For possible lymphatic dysplasia, lymphoscintigraphy and biochemical investigation (lipid profile) of ascitic or pleural fluid were carried out. Suspected inherited metabolic disorders were screened with plasma ammonia, serum lactate, urine organic acid, and plasma amino acid analysis. Toxoplasmosis, rubella, CMV, and herpes simplex were all tested for intrauterine illnesses in all of the babies. In a small number of undiagnosed instances of non-immune HF, modest genetic testing in the form of karyotyping may be possible.

The clinical and demographic characteristics of infants with non-immune HF who died and those who survived were compared. All indicators strongly associated with mortality were incorporated in stepwise multiple logistic regression models to establish independent predictors of death.

## Results

During the 10-year study period, 33 cases of non-immune HF were detected in our tertiary care perinatal referral center from 53,033 live-born neonates, with an incidence of 0.62/1,000 live births.

The median gestational age was 35 years (range: 32-36 years), whereas the mean birth weight was 2.34 ± 0.72 kg. Seventeen (52%) were products of consanguineous marriage. Prenatal diagnosis of non-immune HF was made in 31 (94%) neonates. Regarding the postnatal procedures, 14 (42%) patients had thoracocentesis, three (9%) had a blood transfusion, and only one (3%) had paracentesis. Twenty-four (73%) neonates presented with effusion in two or more serous cavities, five (15%) with pleural effusion, and four (12%) with ascites only. At birth, 22 (67%) neonates required aggressive resuscitation either by positive pressure ventilation (PPV), chest compression (CC), or a lifesaving drug (epinephrine). Twenty-two (67%) infants needed mechanical ventilation while 21 (64%) of them had moderate to severe hypoxic respiratory failure (HRF) with an oxygenation index (OI) of >14. Among the total 33 total cases, 22 (67%) died while 11 (33%) survived. The demographic and clinical profiles of neonates with non-immune HF are shown in Table 1.

**Table 1:** Demographic and clinical profile of neonates with non-immune hydrops fetalis (n=33) SD: standard deviation; FOC: fronto-occipital circumference; HF: hydrops fetalis; LSCS: lower segment cesarean section; SVD: spontaneous vaginal delivery; PPV: positive pressure ventilation; CC: chest compression; OI: oxygenation index; HFOV: high-frequency oscillatory ventilation

Characteristics	Values
Gender (male/female), n (%)	20/13 (61%/39%)
Gestational age (weeks), median (range)	35 (32–36)
Weight (kg), mean ± SD	2.34 ± 0.72
Height (cm), mean ± SD	45.03 ± 4.96
FOC (cm), mean ± SD	32.98 ± 2.62
Place of delivery (inborn/outborn), n (%)	30/3 (91%/9%)
Consanguinity, n (%)	17 (52%)
Prenatal diagnosis of HF, n (%)	31 (94%)
Postnatal diagnostic and therapeutic procedure, n (%)	
Thoracocentesis	14 (42%)
Blood transfusion	3 (9%)
Paracentesis	1 (3%)
Mode of delivery (LSCS/SVD), n (%)	29/4 (88%/12%)
APGAR score (5 minutes), mean (range)	7 (3–9)
Resuscitation required at birth (PPV ± CC ± drug), n (%)	22 (67%)
Affected compartments in HF, n (%)	
Ascites	4 (12%)
Pleural effusion	5 (15%)
Two or more serous cavity effusion	24 (73%)
Hypoxic respiratory failure (moderate to severe OI >14), n (%)	21 (64%)
Need for mechanical ventilation, n (%)	22 (67%)
Need for HFOV, n (%)	7 (21%)
Need for surfactant, n (%)	3 (9%)
Pneumothorax, n (%)	4 (12%)
Duration of mechanical ventilation (days), median (range)	3 (1–7)
Duration of hospitalization (days), median (range)	6 (1–13)
Died, n (%)	22 (67%)
Survived, n (%)	11 (33%)

Among the neonates with non-immune HF, a credible cause could be identified in 17 (51.5%) cases. Cardiovascular disease was the most commonly found (five, 15.1%) underlying cause in neonates with non-immune HF, followed by genetic syndromes (four, 12.1%), and both had 100% mortality as shown in Table 2. There were two cases of severe ventricular dysfunction, one case of hypertrophic cardiomyopathy, one case of dilated cardiomyopathy, and one case of complicated heart disease among the five neonates with cardiovascular diseases. Two of the cases with genetic syndromes had Down syndrome, one had Edwards' syndrome, and one had an unclassified syndrome. Among other identifiable causes, two (6%) had a placental disorder (twin-twin transfusion syndrome) and two (6%) had a renal disorder (posterior urethral valve with vesicoureteral reflux). There was one case (3%) each of hematological (transient abnormal myelopoiesis), lymphatic (chylothorax), infective (congenital rubella syndrome), and neurological disorders (Arnold Chiari malformation II). All of these aforementioned cases survived in our study. However, only six of the 16 (48.4%) cases with unknown etiology survived, while the other 10 died.

**Table 2:** Etiology of non-immune hydrops fetalis (n=33) PDA: patent ductus arteriosus; VSD: ventricular septal defect; ASD: atrial septal defect; CoA: coarctation of the aorta

Determinants	N (%)
Cardiovascular diseases	5 (15.1)
Hypertrophic cardiomyopathy	1 (3)
Dilated cardiomyopathy	1 (3)
Severe ventricular dysfunction	2 (6)
Complex heart disease (PDA + VSD + ASD + CoA)	1 (3)
Genetic syndrome	4 (12.1)
Down syndrome	2 (6)
Edwards' syndrome	1 (3)
Unclassified syndrome	1 (3)
Lymphatic disorder (chylothorax)	1 (3)
Hematologic disease (transient abnormal myelopoiesis)	1 (3)
Placental disorders (twin-twin transfusion syndrome)	2 (6)
Renal disorders (posterior urethral valve with vesicoureteral reflux)	2 (6)
Neurologic malformation (Arnold Chiari malformation II)	1 (3)
Infections (congenital rubella)	1 (3)
Unidentified etiology	16 (48.4)

When comparing newborns who survived with those who died, neonates who died had significantly higher requirements for mechanical ventilation, moderate to severe HRF (OI >14), and prolonged hospitalization (p=0.027, 0.001, and 0.048, respectively). The effects of gestational age and the presence of two or more serous cavity effusions were not statistically significant in our study (Table 3).

On stepwise multiple logistic regression analysis, the need for mechanical ventilation, prolonged hospitalization, and the need for surfactant therapy were found to be independent factors associated with mortality (Table 4).

**Table 3:** Effect and association of study outcomes and factors affecting the mortality of the patients *Statistically significant HF: hydrops fetalis; HRF: hypoxic respiratory failure; OI: oxygenation index; HFOV: high-frequency oscillatory ventilation; IQR: interquartile range; SD: standard deviation

Factors	Description	Outcome		P-value
		Died (n=22)	Survived (n=11)	
Gender	Male, n (%)	12 (55%)	8 (73%)	0.391
	Female, n (%)	10 (45%)	3 (27%)	
Gestational age (weeks)	Mean ± SD; median (IQR)	33.91 ± 3.13	34 (33–36)	0.931
Place of delivery	Inborn, n (%)	20 (91%)	10 (91%)	0.999
	Outborn, n (%)	1 (9%)	2 (9%)	
Consanguinity	Yes, n (%)	13 (59%)	4 (36%)	0.224
	No, n (%)	9 (41%)	7 (64%)	
Diagnosis	Prenatal, n (%)	21 (95%)	10 (91%)	0.631
	Postnatal, n (%)	1 (5%)	1 (9%)	
Postnatal diagnostic and therapeutic procedure	Thoracocentesis, n (%)	10 (45%)	4 (36%)	0.782
	Blood transfusion, n (%)	1 (5%)	2 (18%)	0.301
	Paracentesis, n (%)	1 (5%)	0 (0.0%)	-
Mode of delivery	LSCS, n (%)	19 (86%)	10 (91%)	0.999
	SVD, n (%)	3 (14%)	1 (9%)	
APGAR score (5 minutes)	Mean ± SD	6.13 ± 2.45	7.90 ± 1.91	0.081
Resuscitation required at birth	Yes, n (%)	18 (82%)	4 (36%)	0.014
	No, n (%)	4 (18%)	7 (64%)	
Affected compartments in HF	Ascites, n (%)	3 (14%)	1 (9%)	0.742
	Pleural effusion	3 (14%)	2 (18%)	0.776
	Two or more serous cavity effusion, n (%)	16 (73%)	8 (73%)	-
Moderate to severe HRF (OI >14)	Yes, n (%)	19 (86%)	2 (18%)	0.001*
	No, n (%)	3 (14%)	9 (82%)	
Need for mechanical ventilation	Yes, n (%)	17 (77%)	5 (45%)	0.027*
	No, n (%)	5 (23%)	6 (55%)	
Need for HFOV	Yes, n (%)	6 (47%)	1 (9%)	0.252
	No, n (%)	16 (73%)	10 (91%)	
Need for surfactant	Yes, n (%)	1 (5%)	2 (18%)	0.232
	No, n (%)	21 (95%)	9 (82%)	
Pneumothorax	Yes, n (%)	3 (14%)	1 (9%)	0.780
	No, n (%)	19 (86%)	10 (91%)	
Duration of mechanical ventilation (days)	Median (IQR)	1.50 (1.00–6.50)	7.00 (7.00–16.00)	0.073
Duration of hospitalization (days)	Median (IQR)	1.00 (1.00–7.00)	13.00 (10.00–16.00)	0.048*

**Table 4:** Identification of predictors of mortality in non-immune hydrops fetalis by multiple logistics regression analysis *Statistically significant AOR: adjusted odds ratio; CI: confidence interval

Factors	AOR [95% CI]	P-value
Need for mechanical ventilation	16.83 [1.37–206.09]	0.027*
Need for surfactant	0.04 [0.001–1.10]	0.057
Duration of hospitalization	0.89 [0.79–0.99]	0.038*

## Discussion

According to recent data from affluent countries, the majority of neonates with HF have non-immune HF, with the prevalence of immune HF reduced to 10%. This has been made possible because of the use of anti-D immunoglobulin prophylaxis, intrauterine transfusions, and rigorous fetal observation of rhesus isoimmunized pregnancies [5]. However, the incidence of immune HF is still considered to be high in lower-middle-income countries (LMICs), but our findings contradict earlier studies, as most of the cases we found in our study had non-immune HF [11]. The standardized quality care in our tertiary care facility and increased awareness of anti-D prophylaxis may explain these findings.

Many pregnancies with immune HF and non-immune HF are referred to our hospital, which is a tertiary care, perinatal referral center for high-risk pregnancies, from all across the country. Because the majority of these pregnant women are referred late in their pregnancy, diagnostic and therapeutic options are often limited. Furthermore, a lack of diagnostic and therapeutic fetal interventions in our setting is thought to be a major contributor to increased mortality in newborns with non-immune HF. The incidence of non-immune HF varies greatly in the literature, ranging from 0.3/1,000 to 2.4/1,000 live births [12,13]. In our study, the incidence of non-immune HF was 0.62/1000 live births, which is within the range of previous research.

The two most common diagnoses associated with non-immune HF in our study were cardiovascular and genetic syndromes. The prognosis was not favorable in all nine neonates with these conditions, including five (15.1%) with structural cardiovascular anomalies and four (12.1%) with genetic syndromes, as all of them died. Other significant causes included placental (twin-twin transfusion syndrome), lymphatic (chylothorax), renal (posterior urethral valve), infectious (congenital rubella), hematological (transient abnormal myelopoiesis), and neurologic causes (Arnold Chiari malformation II).

As per the recent studies, there is significant variability with respect to unidentified cases in non-immune HF, ranging from 4-60%. It depends primarily on the availability of resources required for extensive workup in diagnosing non-immune HF [3,14,15] In our study, we found a large number of cases (16, 48%) classified as those with unidentified etiology. This may be due to the limitation of diagnostic facilities available in our setup. Another explanation of the high occurrence of idiopathic reasons may be the limited amount of time available to reach an exact diagnosis, as the majority of newborns with non-immune HF died during the first week of life. Furthermore, the lack of postmortem examinations for idiopathic non-immune HF cases added to the difficulty in determining the cause. Rodrguez et al. have verified this, with postmortem investigation confirming the cause of HF in 51 stillborn fetuses, which amounted to 92% of the cases.

In the literature, the incidence of inborn errors of metabolism (IEM) has been calculated to be 1-2% of all non-immune HF [1]. Although we could not document any case with IEM, we assume the incidence may be high due to the highly prevalent practice of consanguineous marriages in Pakistan.

Studies have revealed several postnatal prognostic indicators in infants with HF [6,10,16,17]. Prenatal HF diagnosis, gestational age, first- and fifth-minute APGAR scores, advanced resuscitation, two or more serous cavity effusions, pleural effusion, and the need for thoracentesis are factors that should be taken into account.

In various studies, gestational age at delivery and two or more serous compartment effusion were found to be important prognostic factors [16,18]. However, these were not found to be statistically significant in our study. This may be attributed to the small sample size of our study. Unlike other reported studies, the need for mechanical ventilation required for HRF and prolonged hospitalization were identified as independent predictors of mortality in our study.

Previously, early delivery for the fetus with HF was associated with favorable outcomes [19]. This has been nullified by a recent publication of large multicenter data, showing a clear association between prematurity and poor outcomes, which can be rationalized with established complications of prematurity. Prolonging the pregnancy with adequate tocolysis, antenatal steroids, and close fetal surveillance is the right approach in these circumstances. However, the administration of antenatal steroids does not influence the overall survival in a fetus with HF [6,20].

Neonates with inadequate response to resuscitation in the delivery room have been associated with increased mortality in the previously published literature [6,16]. These findings are in line with our research, which found that fatal cases necessitated extensive and advanced resuscitation in the delivery room and had considerably low APGAR scores in the first minute.

Rustico et al. have shown in their study that intrauterine pleural drainage may improve the outcomes in fetuses with persistent pleural effusion [21]. This prevents fatal complications associated with pulmonary hypoplasia by decompressing the lungs and heart and thereby providing the optimum chance for the fetus to survive. It is assumed that thoracentesis alone is of limited usefulness since pleural fluid reaccumulates within seven days of the surgery. As a result, it was established that thoracoamniotic shunting may enhance survival rates, particularly in fetuses with quickly reaccumulating effusion [9]. We were unable to execute any of these intrauterine operations due to a lack of resources and experience.

## Conclusions

Although the incidence of immune HF has significantly decreased with the widespread use of anti-D immunoglobulins, non-immune HF is still associated with significant mortality. The need for mechanical ventilation (moderate to severe HRF) and prolonged hospitalization are strong predictors of poor outcomes in neonates with non-immune HF. Therefore, severe hydrops causing significant mortality can be anticipated based on the patients' respiratory status and the need for escalated oxygen support.
